# Metal(loid)s and minerals in fish tissues: health risk–benefit assessment and variations by gender and size

**DOI:** 10.1007/s10653-025-02608-4

**Published:** 2025-06-29

**Authors:** Memet Varol, Emel Kaçar, Yasin Polat

**Affiliations:** 1 Aquaculture Engineering Department, Agriculture Faculty, Malatya Turgut Özal University, Malatya, Turkey; 2 Plant and Animal Production Department, Avanos Fine Arts Vocational School, Nevşehir Hacı Bektaş Veli University, Nevşehir, Turkey; 3 Nevşehir Hacı Bektaş Veli University, Science and Technology Application and Research Center, Nevşehir, Turkey

**Keywords:** Metals and minerals, Bioaccumulation, Fish size, Health risk assessment, Nutritional values

## Abstract

In this study, the concentrations of 15 metal(loid)s and minerals (MMs) were measured in the muscle, liver and gills of two economically important fish species (*Cyprinus carpio* and *Tinca tinca*) collected from Hirfanlı Reservoir (Türkiye) using inductively coupled plasma–tandem mass spectrometry. The effects of fish weight, length and gender on the concentrations of MMs in the tissues, the health risks of metal(loid)s and the nutritional importance of essential metals and minerals were also evaluated. Results indicated that MM concentrations were similar between the two species, likely due to their simultaneous capture from the same reservoir location. Generally, higher MM levels were found in the liver and gills than in muscle. The most abundant MMs in the gills, liver and muscle were Ca (9677 mg/kg wet weight (ww)), P (5161 mg/kg ww) and K (5398 mg/kg ww), respectively. No statistically significant trends were observed in MM accumulation in the tissues based on fish gender or size. Both species were good sources of essential nutrients (Ca, Mg, P, K, Zn, Fe, Mn). The average concentrations of As, Cd, Pb, Cr, and Zn were below regulatory safety limits, indicating no significant health risk. Non-carcinogenic and carcinogenic risk values indicated that consuming these fish species would not pose a health risk to adults. Weekly consumption amounts below 210 g for *C. carpio* and below 280 g for *T. tinca* were deemed safe. The findings suggest that the nutritional benefits of these fish outweigh the potential health risks from metal(loid)s.

**Supplementary Information:**

The online version contains supplementary material available at 10.1007/s10653-025-02608-4.

## Introduction

Recent scientific studies report that metal(loid) pollution in surface water resources worldwide has increased due to intensive agricultural and mining activities, industrialization and fast population expansion (Canpolat et al., 2020; Hamidian et al., 2023; Marini et al., 2021). Metal(loid)s can have a toxic effect on aquatic organisms, accumulate in aquatic organisms, and thus reach humans through the food chain and cause health problems (Akila et al., 2022; Biswas et al., 2023; Tokatlı & Varol, 2021).Researchers generally classify metal(loid)s into two groups: essential and non-essential metal(loid)s. Non-essential metal(loid)s such as Cd, As, Hg, and Pb have no known biological functions and can cause harmful effects in humans even at low concentrations (Blankson et al., 2024; Su et al., 2023; Yang et al., 2021). In contrast, essential metal(loid)s such as Cu, Zn, Fe, and Se are vital for various physiological functions and metabolic processes. However, even essential metal(loid)s can be toxic and pose health hazards above a certain concentration (Blankson et al., 2024; Su et al., 2023).

The fact that fish is rich in vitamins, proteins, minerals and omega-3 polyunsaturated fatty acids is an indication of its importance in human nutrition (Kalipci et al., 2023; Luczynska et al., 2022; Sroy et al., 2021). Fish is also the food type containing the highest concentration of some toxic metal(loids) (Varol & Kaçar, 2023). In this respect, people who consume fish containing metal(loids) for a long time may experience serious health problems (Storelli et al., 2020; Töre et al., 2021). However, the health advantages provided by consuming fish should not be neglected (Olmedo et al., 2013; Sofoulaki et al., 2019; Varol & Kaçar, 2023). Despite its risks, it is necessary not to remove fish from the table due to its numerous health benefits, which are essential for a balanced diet. Previous studies have emphasized that the benefits of a diet rich in fish outweigh its disadvantages (Sofoulaki et al., 2019; Varol & Kaçar, 2023).

Fish absorb metal(loid)s and minerals (MMs) primarily through their gills or diet. However, MM concentrations vary widely across different tissues and organs (Kaçar, 2024; Vetsis et al., 2021). For example, there are studies showing that MM concentrations accumulated in the gills and liver are much higher than in the muscle (Kalantzi et al., 2019; Varol & Kaçar, 2023). The following factors contribute to the emergence of this situation: the concentration of MM in the water and food of the fish, the chemical properties of the water, the fish species, the habitat and the physiological conditions of the fish (Ge et al., 2020; Sofoulaki et al., 2018). In addition, the size, weight and gender of the fish can affect the accumulation of some MMs at higher rates in different tissues and organs of fish (Ge et al., 2020; Merciai et al., 2014). Given these variations in MM accumulation, assessing their potential health risks in human consumers becomes crucial. The majority of studies on metal(loid) contamination in fish have focused on the muscle tissue, which is the most ingested part of fish by humans (Ustaoğlu & Yüksel, 2024; Varol et al., 2022a). Unlike most studies that focus solely on muscle tissue, this research examines multiple tissues and considers gender- and size-related accumulation patterns, providing a more comprehensive understanding of MM bioaccumulation.

As is known, consumption of contaminated fish is the main route metal(loid)s pass into people and causes serious health risks for consumers. In this respect, determination of the metal(loid) contents of fish muscle tissue is critical for protecting of human health. Health risk assessment indices have been widely used in recent years to assess the health risks caused by metal(loid)s accumulated in fish muscle and thus to protect public health (Kaçar, 2024; Prabakaran et al., 2024; Ustaoğlu & Yüksel, 2024). In this study, both the health risks of metal(loid)s in fish muscle tissue and the nutritional importance of essential metals and minerals were evaluated.

Despite the extensive research on metal(loid) contamination in fish, limited studies have simultaneously examined MM accumulation across multiple fish tissues, the effects of gender and size, and the balance between metal(loid) toxicity and nutritional benefits. This comprehensive study was conducted to address this gap in the literature and the concentrations of 15 MMs were measured in three tissues (gills, muscle and liver) of two commonly consumed fish species (*Cyprinus carpio* and *Tinca tinca*) taken from the Hirfanlı Reservoir (Türkiye) to achieve the following objectives: (i) to compare MM concentrations between fish species and fish tissues; (ii) to determine the influence of fish weight, length and gender on MM accumulation in tissues; (iii) to evaluate the health risks of metal(loid)s in fish muscle tissue and the nutritional benefits of essential metals and minerals; (iv) to estimate safe fish consumption limits for human health.

## Materials and methods

### Research area and collection of fish samples

One of the largest reservoirs in Türkiye, the Hirfanlı Reservoir is located in the Central Anatolia Region of the country (Fig. S1). Built on the Kızılırmak River in Kırşehir between 1953 and 1959, the reservoir is used for flood control, electricity generation, irrigation and fishing. The area of ​​the reservoir is 263 km^2^, its volume is 5.98 km^3^, its length is 75 km and its widest point is 15 km (Zeybekoglu, 2023).

*Cyprinus carpio* and *Tinca tinca* individuals were obtained from fishermen hunting in the Kaman region of Hirfanlı Reservoir in July 2023. After obtaining 12 individuals for each species, the samples were immediately brought to the laboratory in a cooling tank with an ice pack. In the laboratory, total length, weight and gender of the fish samples were determined (Table S1). Finally, the gills, liver and dorsal muscle of each sample were removed using stainless steel instruments, packaged independently and kept at − 20 °C until analysis (Varol et al., 2020).

### Metal(loid) and mineral analysis and quality control

A total of 72 samples (24 individuals × 3 tissues) were analyzed to determine the concentrations of 15 MMs including Cd, Pb, Mn, Fe, Zn, As, Ni, Cr, Co, Sr, K, Mg, Na, Ca and P. Firstly, approximately 0.5 g (wet weight) of sample was placed into digestion tubes, followed by 7 mL of HNO_3_ and 1 mL of H_2_O_2_. Then, the digestion vessels were processed in a microwave digestion system (Milestone Start D, USA) following the manufacturer’s protocol. After cooling, the digests were transferred to 50 mL polypropylene tubes, and ultrapure water was added to a final volume of 25 mL. The MM concentrations were analyzed using ICP-MS/MS (Agilent 8800, USA). The accuracy of the method was validated using Certified Reference Material (DORM-2), with recovery rates between 90.7% and 109.2% (Table S2). The limits of detection (LOD) for each MM were determined according to standard protocols, and concentrations below the LOD were replaced by LOD/2 in subsequent statistical analyses (Varol et al., 2020).

### Metal(loid) contamination index

The Metal(loid) Contamination Index (MCI) serves as a metric for total metal(loid) contamination in fish, allowing for comparisons of contamination across different species or tissues. It is calculated by taking the geometric mean of the metal(loid)s in each fish tissue. A higher MCI value is a sign of higher levels of metal(loid) pollution in fish species and tissues (Töre et al., 2021). The equation used in the calculation of the MCI is given in Table 1.

**Table 1:** Metal contamination index and health risk assessment indices used in this study (adopted from Varol et al., 2022b)

Indices	Equations	Explanations
Metal contamination index (MCI)	$\text{MCI}=\left(\text{C}_1\times \text{C}_2\dots \times \text{C}_n\right)^{1/n}$	C_1_, C_2_… C_n_ is the concentration (mg/kg, ww) of each metal in fish tissue; n is the total number of metals (*n* = 10 in this study)
Estimated daily intake (EDI)	$\text{EDI}=\frac{\text{C }\times \text{IRd}}{\text{BW}}$	C is the average metal concentration in fish muscle (mg/kg, ww); IRd is average fish consumption rate (20 g/day; GDFA, 2023); BW is average adult body weight (70 kg)
Target hazard quotient (THQ)	$\text{THQ}=\frac{\text{EF}\times \text{ED}\times \text{IRd}\times \text{C}}{\text{RfD}\times \text{BW}\times \text AT_{noncancer}}\times 10^{-3}$	EF is the exposure frequency (350 days/year); ED is the exposure duration (26 years); RfD is the reference dose (mg/kg-day) of each metal; AT_noncancer_ is the averaging time for non-carcinogen metals (365 × 26 days)
Hazard index (HI)	$\text{HI}=\sum_{\text{n}}^{\text{i}}\text{THQ}_n$	HI is the sum of the THQ values of all metals (*n* = 10)
Carcinogenic risk (CR)	$\text{CR}=\frac{\text{EF}\times \text{ED}\times \text{IRd}\times \text{C}\times \text{CSF}}{\text{BW}\times \text AT_{cancer}}\times 10^{-3}$	CSF is the carcinogenic slope factor of inorganik As (1.5 mg/kg-day^−1^); AT_cancer_ is the averaging time for carcinogen metals (365 × 70 days)
Safe fish consumption amount (SFCA)	$\text{SFCA}_{noncancer}=\frac{\text{RfD}\times \text{BW}}{\text{C}}$ $\text{SFCA}_{cancer}=\frac{\text{ARL}\times \text{BW}}{\text{C}\times \text{CSF}}$	ARL is the acceptable lifetime risk level (ARL = 10^–5^)

### Health risk assessment indices

Various indices such as Estimated Daily Intake, Target Hazard Quotient, Safe Fish Consumption Amount and Carcinogenic Risk were employed to assess the health risks for adults arising from metal(loid) content in fish muscle. In the calculation of these indices, the inorganic form of As was used and assumed toaccount for 3% of the total As (Sofoulaki et al., 2019; Varol & Kaçar, 2023). The equations and explanations used in the calculation of all health risk assessment indices are presented in Table 1.

To estimate the risk of consumers’ exposure to non-carcinogenic effects of metal(loid)s in fish muscle, the estimated daily intake (EDI) values ​​(μg/kg body weight/day) of 10 metal(loid)s were computed and compared with the reference doses (RfDs) set by USEPA (2024).

The target hazard Quotient (THQ) is an index employed to evaluate non-carcinogenic human health risks associated with metal(loid)s in fish muscle through ingestion of fish (Kaçar, 2024). In this study, THQ values were calculated for 10 metal(loid)s with RfD values. The hazard index (HI) is used to assess the combined health risks from exposure to all metal(loid)s examined (Töre et al., 2021). THQ or HI values < 1 suggest that consumers exposed to metal(loid)s in fish muscle are unlikely to face non-carcinogenic risks. Values > 1 suggest that non-carcinogenic health risks may occur in consumers (USEPA, 1989).

According to USEPA (1991), carcinogenic risk (CR) refers the increased probability of developing cancer as a result of exposure to a carcinogenic chemical during a person's lifetime. Since only inorganic arsenic (iAs) is carcinogenic to humans among the metal(loid)s examined in this research (USEPA, 2024), the CR value of iAs was calculated. USEPA (1991) reported that the carcinogenic risk is acceptable when CR values ​​are between 10^–4^ and 10^–6^, that carcinogenic risk is absent when CR values ​​are < 10^–6^, and that the risk is unacceptable when CR values ​​are > 10^–4^.

In order to determine the limits of fish that can be safely consumed over a particular period of time, the Safe Fish Consumption Amount (SFCA) was calculated based on the carcinogenic and non-carcinogenic effects of metal(loids) found in fish muscle (Varol & Kaçar, 2023).

### Nutritional importance of essential metals and minerals

According to the EU Regulation No. 1169/2011 (EU, 2011), a food is considered an important source of minerals if its 100 g contains 15% of the nutrient reference values ​​(NRV). In the present study, the average contribution rates (%) of essential metals (Fe, Mn and Zn) and minerals (Ca, Mg, K and P) detected in the fish muscle to the daily NRVs established for adults by this regulation were calculated. This approach allows for the identification of fish species that provide significant nutritional value while maintaining low toxicity risks.

### Statistical methods

Parametric tests were chosen because MMs showed normal distribution according to the Shapiro–Wilk test. One-way ANOVA was used to compare MM concentrations across different tissues due to its suitability for multiple-group comparisons, while the t-test was applied for species and gender comparisons. Pearson correlation analysis assessed relationships between fish size and MM levels. Statistical analyses in this study were done using Origin 2024b and SPSS 22 programs.

## Results and discussion

Table 2 presents the average concentrations of MMs in the gills, liver, and muscle of *C. carpio* and *T. tinca*. The most abundant minerals in the gills of both species were Ca and P, while P and K were the most abundant minerals in the liver and muscle of both species. The metal(loids) with the highest concentrations in muscle, gills and liver of both species were Zn and Fe, while the metal(loids) with the lowest concentrations in all three tissues were Cd and Co. Similar findings were reported in the muscle, gills and liver of fish species collected from the Atatürk Reservoir (Varol et al., 2022a).

**Table 2:** Metal(loid) and mineral concentrations (mg/kg, ww) in the tissues of both fish species

Species		N		Na	Mg	P	K	Ca	Cr	Mn	Fe	Co	Ni	Zn	As	Sr	Cd	Pb
*C. carpio*																		
	Gills	12	Mean	695^a^	402^a^	6553^a^	2755^a^	7118^a^	0.186	4.08^a^	110.6^a^	0.178^a^	0.054	144.1^a^	1.55	56.6^a^	0.0107	0.240
			S.D	59	161	1804	408	4099	0.222	4.93	146.9	0.139	0.072	44.3	3.28	30.0	0.0245	0.359
	Liver	12	Mean	689^a^	251^b^	4946^b^	3082^a^	439^b^	0.118	2.79^ab^	100.2^a^	0.051^b^	0.096	130.1^a^	0.731	2.58^b^	0.017	0.206
			S.D	126	48	972	436	307	0.043	0.656	17.5	0.061	0.042	61.8	1.709	2.52	0.0177	0.263
	Muscle	12	Mean	318^b^	341^a^	4055^b^	5398^b^	687^b^	0.109	0.419^b^	6.88^b^	0.022^b^	0.056	9.6^b^	0.609	2.17^b^	0.0025	0.099
			S.D	70	28	583	411	403	0.031	0.343	6.97	0.037	0.047	7.2	1.265	2.45	0.0059	0.104
*T. tinca*																		
	Gills	12	Mean	755^a^	528^a^	8472^a^	2208^a^	9677^a^	0.156^a^	2.57^a^	72.0^a^	0.17^a^	0.073	19.1^a^	0.914	85.9^a^	0.0079^ab^	0.177
			S.D	73	143	2220	394	5271	0.067	0.961	23.1	0.067	0.061	3.2	0.921	30.7	0.0054	0.124
	Liver	12	Mean	758^a^	272^b^	5161^b^	3041^b^	337^b^	0.08^b^	2.25^a^	82.8^a^	0.043^b^	0.077	48.4^b^	0.451	2.28^b^	0.0108^b^	0.172
			S.D	128	61	817	436	243	0.024	0.824	33.4	0.038	0.047	32.5	0.684	1.87	0.0108	0.188
	Muscle	12	Mean	312^b^	305^b^	3652^c^	5247^c^	617^b^	0.12^a^	0.297^b^	5.81^b^	0.031^b^	0.064	4.9^a^	0.369	1.76^b^	0.0036^a^	0.115
			S.D	51	39	806	777	535	0.038	0.248	3.80	0.061	0.045	1.6	0.589	2.89	0.0054	0.109

Different letters in the same column represent significant differences between tissues (gills, liver and muscle) for each species (*p* < 0.05)

*N* number of samples, *SD* standard deviation

### Metal(loid) and mineral concentrations in species

The t-test results (Table S3) revealed that the average concentrations of most MMs did not vary statistically between two fish species (*p* > 0.05). Only the average concentrations of Na, P and Sr in the gills and Mg in the muscle of *T. tinca* were higher than in *C. carpio* (*p* < 0.05) (Tables 2 and S3). However, the average concentrations of K and Zn in the gills, Cr and Zn in the liver, and Zn in the muscle of *C. carpio* were higher than in *T. tinca* (*p* < 0.05) (Tables 2 and S3).

Previous studies have reported that MM concentrations in fish tissues show species-specific differences (Sofoulaki et al., 2018; Varol & Kaçar, 2023; Varol et al., 2022a). In the present study, the average concentrations of only two MM differed among the 15 MMs in the muscle and liver of fish species (Table S3). This may be due to the fact that individuals of both species were obtained from the same region of the reservoir at the same time. It was determined that the Zn concentrations in the gills, liver and muscle of *C. carpio* were much higher compared to *T. tinca*. This finding indicated that the Zn content in fish may vary depending on the species. Similarly, previous studies reported that the Zn concentrations detected in different tissues of *C. carpio* were considerably higher than other fish species (Jeng et al., 1999; Sun & Jeng, 1998). The average concentrations of five MMs in the gills of fish species were found to vary significantly, which may be attributed to physiological conditions, habitat preferences, diets, ecological needs and metabolic rates of the species (Jiang et al., 2022; Pan et al., 2022; Vetsis et al., 2021). For instance, the higher concentrations of Na, P and Sr in the gills of *T. tinca* may be due to the fact that it is a demersal fish (Varol et al., 2022b).

### Tissue-specific accumulation

One-way ANOVA results revealed that the average concentrations of MMs except Cr, Ni, As, Cd and Pb in *C. carpio* and Ni, As and Pb in *T. Tinca* displayed statistically significant variations among muscle, liver and gills (*p* < 0.05). As shown in Table 2, the concentrations of MMs recorded in the gills or liver of both species, except for K, were higher than those recorded in muscle tissue. Consistent with this finding, many studies comparing MM concentrations in different fish tissues informed that gills and liver have higher MM contents than muscle tissue (Ge et al., 2020; Pan et al., 2022; Varol & Kaçar, 2023).

In fish, the internal regulatory mechanisms and the functions of various tissues are crucial in MM accumulation (Kalantzi et al., 2019; Pan et al., 2022). For example, the gills and liver are capable of accumulating higher concentrations of MMs compared to other tissues because they are metabolically active organs (Pan et al., 2022; Varol & Kaçar, 2023). In addition, gills are the main target organ exposed to MMs because they are in continual contact with water and filter a lot of water. In this respect, MM concentrations measured in gills provide an idea about MM concentrations in ambient water (Kalantzi et al., 2019; Tokatlı, 2018; Vetsis et al., 2021). The liver, on the other hand, plays a crucial function in both the transport and storage of essential metals through its metal-binding proteins and also acts as a barrier to prevent the harmful effects of metal(loid)s (Ge et al., 2020; Pan et al., 2022; Varol & Kaçar, 2023). However, muscle does not accumulate high concentrations of MMs because its metabolic activity is comparatively lower than that of the liver and gills (Kalantzi et al., 2019; Vetsis et al., 2021). Moreover, MMs in muscle tissue have a higher dilution rate than other organs because since muscle tissue constitutes the majority of a fish’s weight (Varol & Kaçar, 2023).

### Influence of gender on MM accumulation

According to T-test results, the concentrations of all MMs recorded in the muscle and liver of female and male individuals of *C. carpio* did not differ statistically (*p* > 0.05) (Table 3). Among the 15 MMs, only Na was found to vary depending on gender in the gills of *C. carpio* and was observed to be in higher concentration in the gills of male fish (*p* < 0.05). Similarly, the concentrations of all MMs recorded in the muscle of female and male individuals of *T. tinca* were not statistically different (*p* > 0.05) (Table 3). However, only Mg was found to vary depending on gender in the liver of *T. tinca* and was observed to be in higher mean concentration in the liver of female fish (*p* < 0.05). When the gills of *T. tinca* were considered, the concentrations of five MMs (Mg, P, Zn, As and Sr) were found to vary depending on gender. Of these five MMs, the average concentrations of Mg, P, Zn and Sr were recorded to be higher in male fish, while the average concentration of As was recorded to be higher in female fish (*p* < 0.05) (Table 3).

**Table 3:** Mean concentrations of metal(loid)s and minerals in the tissues of male and female fish

Species		N		Na	Mg	P	K	Ca	Cr	Mn	Fe	Co	Ni	Zn	As	Sr	Cd	Pb
*C. carpio*																		
	Muscle	5	Male	308	356	3713	5449	583	0.098	0.41	4.9	0.0408	0.041	7.9	1.333	1.93	0.0056	0.1319
		7	Female	326	331	4298	5361	761	0.117	0.42	8.3	0.0089	0.067	10.8	0.092	2.33	0.0003	0.0762
	Liver	5	Male	669	254	4986	3108	390	0.127	3.03	95.5	0.0718	0.086	151.8	1.614	2.54	0.0239	0.3606
		7	Female	703	249	4918	3064	474	0.111	2.62	103.5	0.0354	0.102	114.6	0.101	2.62	0.0121	0.0954
	Gills	5	Male	734^a^	362	6245	2852	5983	0.142	3.12	80.6	0.1947	0.061	159.2	2.934	46.66	0.0212	0.43
		7	Female	667^b^	430	6772	2686	7928	0.218	4.77	132.0	0.1655	0.050	133.3	0.564	63.74	0.0032	0.1039
*T. tinca*																		
	Muscle	3	Male	333	281	3049	4520	407	0.099	0.41	5.0	0.0378	0.081	5.2	0.016	0.91	0.0008	0.0745
		9	Female	305	312	3853	5489	686	0.127	0.26	6.1	0.0285	0.058	4.9	0.487	2.05	0.0046	0.1288
	Liver	3	Male	701	212^a^	4543	2883	337	0.077	1.67	111.1	0.033	0.045	62.6	0.013	2.22	0.0066	0.1664
		9	Female	777	292^b^	5367	3094	338	0.080	2.44	73.4	0.0469	0.088	43.7	0.598	2.30	0.0122	0.1735
	Gills	3	Male	749	739^a^	11552^a^	1852	16,497	0.162	3.55	70.4	0.149	0.088	22.6^a^	0.133^a^	130.3^a^	0.0034	0.0778
		9	Female	757	458^b^	7446^b^	2327	7403	0.153	2.25	72.5	0.1767	0.068	18.0^b^	1.174^b^	71.2^b^	0.0094	0.2094

Different letters in the same column indicate statistical difference between males and females for each tissue (*p* < 0.05)

Vieria et al. (2011) reported that many factors such as ecological needs, life cycle stage and metabolic activities may contribute to gender-related variations in MM concentrations in fish tissues. However, gender was determined to have no substantial effect on the concentrations of most MMs in the three tissues of the fish species considered in the present investigation. The spawning period of *C. carpio* and *T. tinca* in the study area lasts from early April to late June. In this research, individuals of both species were collected at the end of July when the gonads were in the recovery stage, which may explain the absence of significant differences in most MM levels between male and female fish. Consistent with our findings, Kaçar (2024) also found that most metals did not show significant differences between males and females of two fish species collected from Damsa Dam Lake (Türkiye). Majnoni et al. (2013) noted no important variations in metal contents in tissues of male and female fish caught from Zarivar Wetland in Iran. Varol et al. (2022a) found that gender had an insignificant effect on the concentrations of most MMs in the skin, liver, gills and muscle of fish species caught from Atatürk Reservoir in Türkiye. Similarly, Rajkowska and Protasowicki (2013) found that metal contents in tissues other than the gonad of male and female fish taken from two lakes (Poland) did not show statistically significant differences. The fact that gonads, which are the organ most associated with gender in evaluating the effects of gender on MM accumulation in fish, were not included in this study is a shortcoming.

## Associations between MM concentrations and fish size

Correlations between fish size and MM concentrations in fish tissues are given in Table 4. Considering *T. tinca* between the studied species, P, Ca, As, Sr and Pb in gills were observed to be negatively related to fish length or weight (*p* < 0.05). Whereas, Na, P, K, Mn, Co, As, Cd and Pb in liver and Mg, K, P, Ca, Sr, Pb, Fe and As in muscle were found to be positively related to length or weight (*p* < 0.05). Considering *C. carpio*, none of MMs in the gills were found to be significantly correlated with fish length or weight (*p* > 0.05). However, it was noted that Fe in liver was positively associated with fish length and weight, while Na, K and Fe in muscle were negatively associated with length and weight (*p* < 0.05).

**Table 4:** Correlation matrix showing associations between MMs in tissues and fish size

			Na	Mg	P	K	Ca	Cr	Mn	Fe	Co	Ni	Zn	As	Sr	Cd	Pb
*C. carpio*																	
	Gills	Total length	0.032	− 0.040	− 0.005	− 0.084	− 0.078	0.041	0.029	0.100	0.344	0.033	− 0.097	0.172	− 0.111	0.113	0.057
		Body weight	0.050	0.141	0.163	− 0.314	0.142	0.102	0.104	0.175	0.407	0.130	0.132	0.327	0.094	0.285	0.270
	Liver	Total length	− 0.185	− 0.248	− 0.372	− 0.207	0.286	− 0.219	− 0.168	0.644^b^	0.192	0.237	0.222	0.061	0.070	− 0.105	− 0.036
		Body weight	− 0.209	0.014	− 0.311	− 0.262	0.356	− 0.225	− 0.345	0.733^a^	0.312	0.286	0.288	0.245	0.233	− 0.023	0.185
	Muscle	Total length	− 0.443	− 0.118	0.172	− 0.203	0.405	0.033	0.198	− 0.491	− 0.024	− 0.297	0.089	0.056	0.466	0.060	0.213
		Body weight	− 0.512^b^	-0.342	− 0.135	− 0.547^b^	0.033	0.066	0.043	− 0.602^b^	0.142	− 0.225	0.203	0.243	0.037	0.150	0.422
*T. tinca*																	
	Gills	Total length	− 0.097	− 0.386	− 0.534^b^	0.425	− 0.507^b^	− 0.405	− 0.225	− 0.163	0.060	0.357	− 0.412	0.330	− 0.509^b^	− 0.073	0.593^b^
		Body weight	0.275	− 0.383	− 0.499^b^	0.399	− 0.490	− 0.430	− 0.332	− 0.043	− 0.172	0.062	− 0.199	0.634^b^	− 0.428	0.110	0.695^a^
	Liver	Total length	0.553^b^	0.464	0.653^b^	0.720^a^	− 0.187	0.421	0.560^b^	0.140	0.659^a^	0.352	0.249	0.730^a^	− 0.157	0.653^b^	0.666^a^
		Body weight	0.451	0.172	0.401	0.574^b^	− 0.281	0.326	0.283	− 0.022	0.349	0.518^b^	0.088	0.716^a^	− 0.226	0.644^b^	0.565^b^
	Muscle	Total length	− 0.453	0.642^b^	0.431	0.543^b^	0.630^b^	− 0.284	− 0.116	0.572^b^	0.105	− 0.445	0.260	0.543^b^	0.629^b^	0.464	0.479
		Body weight	− 0.460	0.700^a^	0.511^b^	0.546^b^	0.581^b^	− 0.302	− 0.200	0.339	0.295	− 0.268	0.222	0.786^a^	0.613^b^	0.381	0.569^b^

^a^Correlation is significant at the 0.01 level

^b^Correlation is significant at the 0.05 level

When the correlation results of both species were compared, it was found that more MMs were associated with fish size in *T. tinca*. This is probably due to the narrower range of both length and weight in *C. carpio* specimens. The fact that the length and weight of *T. tinca* specimens exhibited higher standard deviation values than *C. carpio* supports this finding. Therefore, it can be said that fish length and weight have no effect on the accumulation of MMs in the tissues of *C. carpio*. On the other hand, it was noted that some of significant associations between fish size and MM concentrations in tissues of both fish were positive, while some were negative. This finding contradicted the findings of Ge et al. (2020) and Merciai et al. (2014) reporting negative associations between MM concentrations in tissues and fish size. Thus, these findings showed that the associations between the contents of MMs in the tissues of both fish species and fish length or weight were unclear and inconsistent.

### Metal(loid) contamination index

**Fig. 1:**
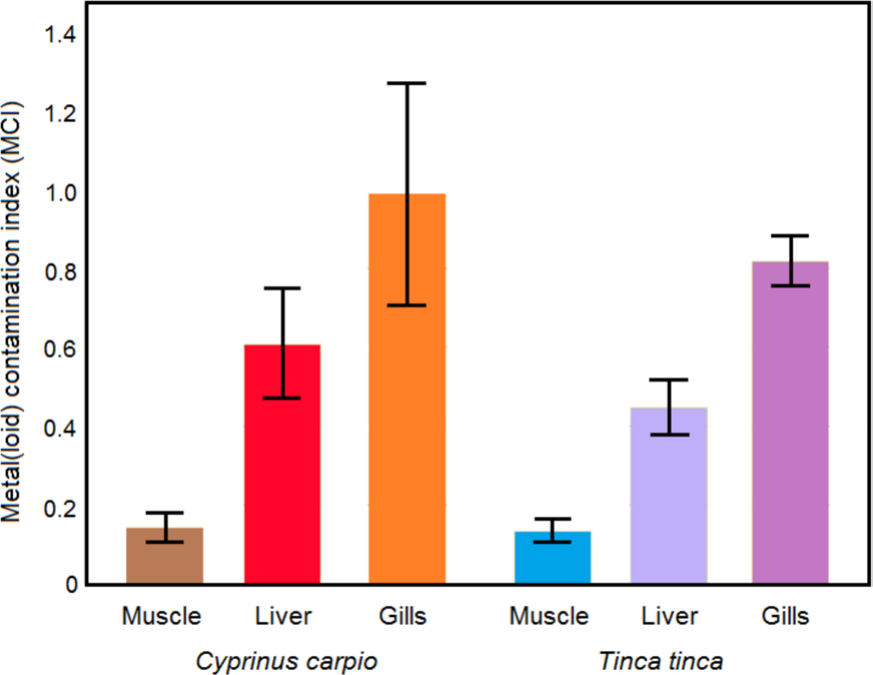
MCI values (mean ± SE) calculated for tissues of each fish species

When the metal(loid) contamination index (MCI) results computed for three tissues of both fish species were examined, it was determined that the MCI values ​​were higher in all three tissues of *C. carpio* than in *T. tinca* (Fig. 1). In addition, the MCI values ​​in both fish species were ranked as gills > liver > muscle (Fig. 1). Assuming that the fish species or fish tissue with the higher MCI value is more contaminated, it can be said that *C. carpio* accumulated more total metal than *T. tinca*, while the gills accumulated more total metal(loid) than the other two tissues. Consistent with our findings, Varol et al., (2022a, 2022b) reported that *C. carpio* had the higher MCI value among the fish species caught from the Atatürk Reservoir and Kızılırmak River and that gills had the higher MCI value among the tissues examined.

### Health risk implications

The mean inorganic As (iAs), Cd, Pb, Cr and Zn concentrations detected in fish muscle were compared with the maximum limits determined by both national and international guidelines to protect consumer health. As shown in Table 5, the mean iAs, Cd, Pb, Cr and Zn concentrations of both species were below the maximum limits. Similarly, the metal(loid) concentrations of fish species caught from other reservoirs and lakes in Türkiye were found below the maximum limits (Kaçar, 2024; Ustaoğlu & Yüksel, 2024; Varol & Sünbül, 2020; Varol et al., 2022a).

**Table 5:** Mean concentrations of metal(loid)s detected in muscle of fish species in this study and maximum permissible limits for fish (units mg/kg ww)

	iAs	Cd	Cr	Zn	Pb	
*C. carpio*	0.0183	0.0025	0.109	9.6	0.099	This study
*T. tinca*	0.0111	0.0036	0.12	4.9	0.115	This study
*Maximum permissible limits*						
Chinese Health Ministry	0.1		2.0			MHPRC (2013)
European Commission Regulation		0.05			0.3	EC (2006)
Food and Agriculture Organization				30		FAO (1983)

iAs: inorganic As (3% of total arsenic)

When the EDI values ​​calculated for metal(loid)s in fish species were examined, it was seen that Fe, Sr and Zn had higher values, while Cd, Co and iAs had lower values (Table 6). However, it was noted that all EDI values were significantly lower than the reference dose (RfD) values ​​set by USEPA (2024) (Table 6). This finding showed that the daily intakes of metal(loid)s in the muscle tissue of both species would not result in health problems for consumers. Similarly, the EDI values of metals in fish species from Dongting Lake (China) were found to be lower than the RfD values (Jiang et al., 2022).

**Table 6:** The results of health risk assesment indices

	**Target Hazard Quotient (THQ)**										**HI**	**CR**
	Cr	Mn	Fe	Co	Ni	Zn	iAs	Sr	Cd	Pb		
***C. carpio***	1.99E-05	8.19E-04	2.69E-03	2.02E-02	7.71E-04	8.74E-03	1.67E-02	9.89E-04	6.79E-04	1.82E-02	6.98E-02	2.79E-06
***T. tinca***	2.19E-05	5.80E-04	2.27E-03	2.82E-02	8.72E-04	4.52E-03	1.01E-02	8.04E-04	9.93E-04	2.11E-02	6.94E-02	1.69E-06

	**Contribution of THQ of each metal to HI (%)**											
	**Cr**	**Mn**	**Fe**	**Co**	**Ni**	**Zn**	**iAs**	**Sr**	**Cd**	**Pb**		
***C. carpio***	0.03	1.17	3.86	29.00	1.11	12.52	23.91	1.42	0.97	26.02		
***T. tinca***	0.03	0.84	3.28	40.56	1.26	6.51	14.57	1.16	1.43	30.33		

	**Estimated Daily Intake (μg/kg bw/day)**											
	**Cr**	**Mn**	**Fe**	**Co**	**Ni**	**Zn**	**iAs**	**Sr**	**Cd**	**Pb**		
***C. carpio***	3.11E-02	1.20E-01	1.96E + 00	6.33E-03	1.61E-02	2.73E + 00	5.22E-03	6.19E-01	7.08E-04	2.84E-02		
***T. tinca***	3.42E-02	8.47E-02	1.66E + 00	8.81E-03	1.82E-02	1.41E + 00	3.16E-03	5.03E-01	1.04E-03	3.29E-02		
**RfD** **(μg/kg bw/day)**	1500	140	700	0.3	20	300	0.3	600	1	1.5		

	**Safe fish consumption amount (SFCA**_**noncancer**_**) (kg fish ww/day)**										**SFCA**_**cancer**_** (kg fish ww/day)**	
	**Cr**	**Mn**	**Fe**	**Co**	**Ni**	**Zn**	**iAs**	**Sr**	**Cd**	**Pb**	**iAs**	
***C. carpio***	963.5	23.4	7.1	0.9	24.9	2.2	1.1	19.4	28.2	1.1	0.03	
***T. tinca***	876.3	33.0	8.4	0.7	22.0	4.2	1.9	23.8	19.3	0.9	0.04	

It was determined that the THQ values ​​of all metal(loid)s in both *C. carpio* and *T. tinca* did not exceed the critical value of 1 (Table 6). Similarly, both species’ calculated HI values were found to be less than 1 (Table 6). These findings suggest that the likelihood of non-carcinogenic health effects in people from the intake of a single metal(loid) or all metal(loid)s found in the fish muscle is low. Similar to our findings, Varol et al. (2022b) found that the THQ and HI values ​​of metal(loid)s in *C. caripo* and *T. tinca* caught from the Kızılırmak River feeding the Hirfanlı Reservoir were less than 1. However, Tokatli (2022) reported that the THQ values of As and Cr and HI values of metal(loid)s in fish species from Gala Lake (Türkiye) exceeded the critical value of 1.

The results indicated that Co, Pb and iAs had the highest THQ values in both fish species, while Cr, Cd and Ni in *C. carpio* and Cr, Mn and Sr in *T. tinca* had the lowest THQ values. While the contributions of Co, Pb and iAs to the calculated HI value for *C. carpio* were found to be 29%, 26.02% and 23.91%, respectively, for *T. tinca* they were found to be 40.56%, 30.33% and 14.57%, respectively (Table 6).

Carcinogenic risk (CR) values ​of iAs, which is assumed to represent 3% of the total As detected in both species, were also calculated. While the CR value for *C. carpio* was found to be 2.79 × 10^–6^, it for *T. tinca* was found to be 1.69 × 10^–6^ (Table 6). The CR values ​​found for both fish species are within the acceptable range (1 × 10^–4^ to 1 × 10^–6^), indicating that consumption of these species will not pose a carcinogenic health risk to humans. However, Hasan et al. (2022) reported that As contents in fish species from a river in Bangladesh had carcinogenic effects.

Safe fish consumption amount (SFCA) refers to the amount of fish (kg) that a 70 kg adult can safely eat in a day without being exposed to health risks from metal(loid)s in fish. In this investigation, it was found that the SFCA values ​​computed for the non-carcinogenic effects of metal(loid)s in both fish species were sufficiently high (Table 6). It was determined that Co had the lowest SFCA values ​​among the metal(loid)s analysed in both species. Considering the lowest SFCA value, it is estimated that non-carcinogenic effects would not occur if an adult weighing 70 kg consumes no more than 900 g of *C. carpio* and 700 g of *T. tinca* per day (Table 6). These SFCA_noncancer_ were found to be lower than those of *C. caripo* and *T. tinca* caught from the Kızılırmak River (Varol et al., 2022b).

The SFCA values ​​calculated for the carcinogenic effects of iAs in fish species were found to be 30 g for *C. carpio* and 40 g for *T. tinca*, respectively (Table 6). It was observed that both SFCA_cancer_ and SFCA_noncancer_ values ​​calculated for fish species exceeded the average daily fish consumption of 20 g reported for adults in Türkiye. According to this finding, non-carcinogenic and carcinogenic risks would not be expected for people consuming both fish species.

USEPA (2000) suggests using more conservative SFCA values ​​to protect consumer health. In this study, SFCA_cancer_ values ​​were determined to be lower than SFCA_noncancer_ values. Therefore, we recommend that a 70 kg adult consume less than the SFCA_cancer_ values, i.e. less than 280 g of *T. tinca* or 210 g of *C. carpio* per week, to maintain health. Overconsumption of contaminated fish may pose health risks, especially for vulnerable populations like pregnant women and children (Waichman et al., 2025). To minimize potential health risks, sensitive groups should limit their intake to levels below the SFCA_cancer_ values established for both fish species.

In this research, only fish consumption was considered in determining the health risks associated with the intake of metal(loid)s. However, it should be noted that there are other sources of metal(loid) exposure, such as the consumption of other foodstuffs and environmental exposures that are not included in this research. In this regard, we recommend ongoing monitoring of metal(loid)s in vegetables, fruits, cereals, fish and other animal food products to evaluate potential health risks in the study area.

### Nutritional evaluation

The contributions of Mg, P, K, Ca, Mn, Fe and Zn detected in fish muscle to the Nutrient Reference Values ​​(NRV) are presented in the Table 7. Among these MMs, the contributions of Mn and Fe were found to be the lowest in both fish species. The contributions of Mn and Fe to the recommended daily intakes were calculated as 2.1% and 4.9% in *C. carpio* and 1.5% and 4.2% in *T. tinca*, respectively. On the other hand, P (52.2–57.9%) was found to contribute the most to the NRVs in both fish species, followed by K (26.2–27%). Additionally, it was determined that Zn, Mg and Ca met 4.9–9.6%, 8.1–9.1% and 7.7–8.6% of the recommended daily intakes, respectively. These results indicated that *C. carpio* contributes more to the dietary requirements of all these MMs than* T. tinca.*

**Table 7:** Contributions of some MMs in fish species to the daily reference intakes

	Contribution to the nutrient reference values (NRVs) (%)						
	Ca	K	Mg	P	Fe	Mn	Zn
*C. carpio*	8.6	27.0	9.1	57.9	4.9	2.1	9.6
*T. tinca*	7.7	26.2	8.1	52.2	4.2	1.5	4.9
NRVs (mg/100 g) (EU, 2011)	800	2000	375	700	14	2	10

The nutritional assessment results showed that both species were good dietary sources of Zn, Fe, Mn, P, K, Ca and Mg. The fact that P and K contributed more than 15% to the relevant NRVs in both fish species suggested that consuming both species is beneficial for a healthy diet. Consistent with our findings, many studies have reported that fish is a rich source of P and K (Ustaoğlu and Yüksek, 2024; Varol and Kaçar, 2022a and 2022b). In addition, it should not be forgotten that in addition to these minerals, fish has an important place in human nutrition in terms of essential fatty acids, vitamins and proteins (Sofoulaki et al., 2019; Ustaoğlu and Yüksek, 2024).

Many studies have emphasized the importance of considering both the nutritional benefits and risks associated with toxic metal(loid) exposure when consuming fish (Sroy et al., 2021; Varol et al., 2022a; Yuvka et al., 2025). This research has provided valuable insights into balancing the nutritional benefits and health risks of fish. It has identified safe consumption limits that aim to minimize health risks while optimizing benefits. Future studies should focus on determining safe consumption limits for all age groups to protect consumer health.

## Conclusions

This research paper offers an in-depth analysis of the concentrations of 15 MMs found in the muscle, liver, and gills of two fish species from Hirfanlı Reservoir in Türkiye. What makes this study unique is its combined evaluation of health risks and nutritional advantages, along with an analysis based on gender and size, utilizing ICP-MS/MS for precise data. By examining various tissues and investigating the biological factors that influence metal(loid) accumulation, this research provides important insights for public health safety, environmental policy, and a better understanding of metal(loid) distribution in aquatic ecosystems. The results indicated that the nutritional advantages of consuming fish surpass the potential health risks, making significant contributions to the fields of environmental chemistry and ecotoxicology.

The findings indicated that gender does not significantly influence the accumulation of most MMs in the tissues of both *Cyprinus carpio* and *Tinca tinca*. The concentrations of all MMs in the muscle and liver of both male and female specimens were not significantly different. There were only a few specific cases where gender-related differences were observed. For instance, in *T. tinca*, males had higher concentrations of Mg, P, Zn, and Sr in the gills, while females had higher levels of As. In *C. carpio*, the only significant gender difference was in the Na concentration in the gills, which was higher in males. According to the correlation analysis results, only a few MMs in the tissues of *C. carpio* were found to be related to fish size, while more MMs were found to be related to fish size in *T. tinca*. However, some of the significant correlations were positive while others were negative, indicating that the relationships between fish size and MM concentrations were not clear.

## Supplementary Information

Below is the link to the electronic supplementary material.

Supplementary file1 (DOCX 864 KB)

## Data Availability

No datasets were generated or analysed during the current study.
