# Supplementary material for: Metal(loid)s and minerals in fish tissues: health risk–benefit assessment and variations by gender and size
Source: Environ Geochem Health. 2025 Jun 29;47(8):290. doi: 10.1007/s10653-025-02608-4 (PMC12206678; doi:10.1007/s10653-025-02608-4)
Supplement: Supplementary file 1 — Supplementary file1 (DOCX 864 KB) [file 10653_2025_2608_MOESM1_ESM.docx]

**Table S1.** Body weight and total length of fish species from the Hirfanlı Reservoir

|  | N | Weight (g) | Total length (mm) |
| --- | --- | --- | --- |
| *Cyprinus carpio* | 12 | 408 ± 35 | 298 ± 11 |
| *Tinca tinca* | 12 | 318 ± 76 | 278 ± 20 |

N, number of samples

**Table S2.** Concentrations of reference material DORM-2,) measured concentrations, recoveries and limits of detection (LOD) for metals and elements in this study

|  | **Na** | **Mg** | **P** | **K** | **Ca** | **Cr** | **Mn** | **Fe** | **Co** | **Ni** | **Zn** | **As** | **Sr** | **Cd** | **Pb** |
| --- | --- | --- | --- | --- | --- | --- | --- | --- | --- | --- | --- | --- | --- | --- | --- |
| Certified values (mg/kg) for DORM-2 | - | - | - | - | - | 34.7 | 3.66 | 142 | 0.182 | 19.4 | 25.6 | 18 | - | 0.043 | 0.065 |
| Measured values (mg/kg) | - | - | - | - | - | 33.8 | 3.75 | 144 | 0.165 | 20.1 | 25.2 | 17.5 | - | 0.046 | 0.071 |
| Recovery (%) | - | - | - | - | - | 97.4 | 102.5 | 101.4 | 90.7 | 103.6 | 98.4 | 97.2 | - | 107.0 | 109.2 |
| ***LOD (mg/kg)*** | 0.008 | 0.006 | 0.03 | 0.06 | 0.02 | 0.0008 | 0.002 | 0.003 | 0.0004 | 0.001 | 0.002 | 0.0006 | 0.002 | 0.0002 | 0.0004 |

**Table S3.** T test results showing differences in MM concentrations between two fish species for each tissue

|  | | N | Na | Mg | P | K | Ca | Cr | Mn | Fe | Co | Ni | Zn | As | Sr | Cd | Pb |
| --- | --- | --- | --- | --- | --- | --- | --- | --- | --- | --- | --- | --- | --- | --- | --- | --- | --- |
|  | Gills | 24 | *** | nd | *** | *** | nd | nd | nd | nd | nd | nd | *** | nd | *** | nd | nd |
|  | Liver | 24 | nd | nd | nd | nd | nd | *** | nd | nd | nd | nd | *** | nd | nd | nd | nd |
|  | Muscle | 24 | nd | *** | nd | nd | nd | nd | nd | nd | nd | nd | *** | nd | nd | nd | nd |

***Significant differences among three fish species for each tissue (*p* < 0.05)

nd: no difference (*p* > 0.05)


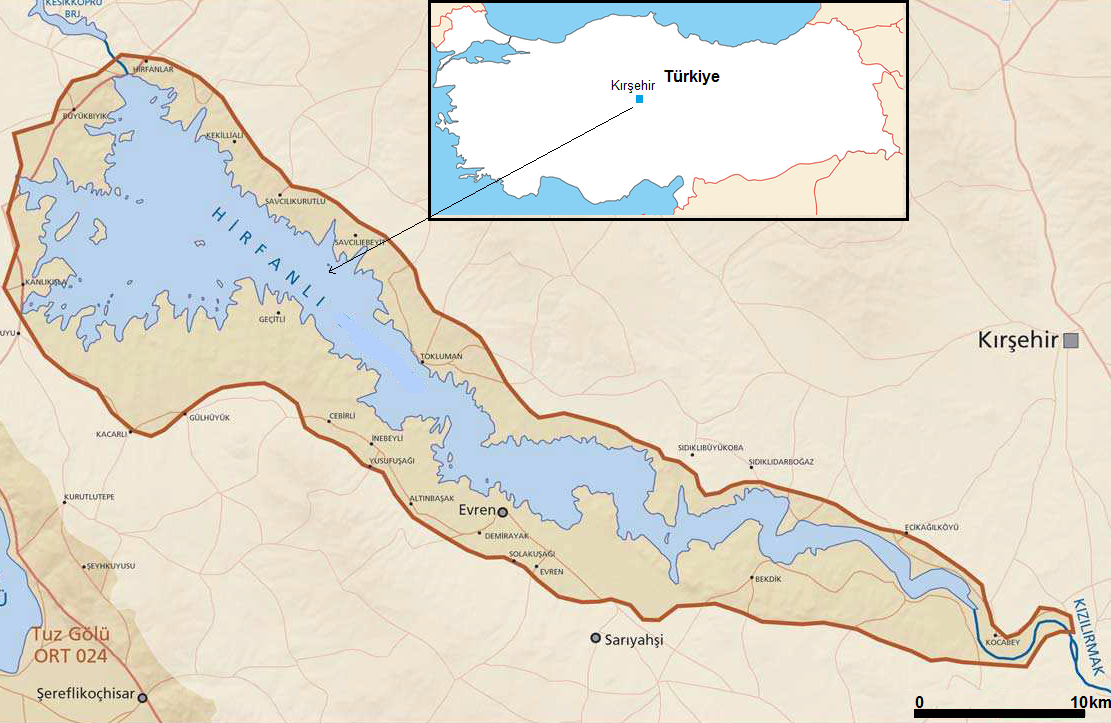


**Fig. S1.** Map of the Hirfanlı Reservoir in Türkiye
